# A Rare Presentation of Posterior Cerebral Artery Stroke With Monocular Hemianopia After Diesel Exposure

**DOI:** 10.7759/cureus.14977

**Published:** 2021-05-12

**Authors:** Nicolette Natale, Ryan Petit, James Champane, Camilo Rodriguez, Julia Ladna, Cristina Savu

**Affiliations:** 1 Internal Medicine, Nova Southeastern University Dr. Kiran C. Patel College of Osteopathic Medicine, Fort Lauderdale, USA; 2 Internal Medicine, American University of the Caribbean, Fort Lauderdale, USA; 3 Internal Medicine, Broward Health Medical Center, Fort Lauderdale, USA

**Keywords:** occipital lobe infarction, diesel exposure, monocular hemianopia, posterior cerebral artery stroke, diesel exhaust, ischemic stroke

## Abstract

Acute ischemic stroke of the posterior cerebral artery (PCA) presents with variable symptoms that may initially make it challenging to diagnose. Common etiologies of PCA stroke include large and small artery disease, atherosclerosis, and cardioembolism. We present a 69-year-old male, initially diagnosed with sinusitis at an urgent care facility, who presented with worsening headache and peripheral vision loss following exposure to diesel vapor and exhaust. Physical examination revealed a right monocular temporal hemianopia and subsequent imaging showed infarction of the left occipital lobe. Due to the length of time between the onset of the infarct and medical treatment, angiography and physical intervention were not indicated and management was done medically. This case presents a unique exposure prior to the development of a PCA stroke, as well as an atypical visual defect and suggests that physicians should consider neuroimaging in patients with nonspecific neurological findings such as new-onset headache and vision changes.

## Introduction

A cerebrovascular accident, also called a stroke, is a neurologic injury that is caused by decreased blood flow to a certain region of the brain that is due to a vascular cause. It can be categorized into stroke if symptoms last more than 24 hours and there are findings on brain imaging suggestive of infarction or hemorrhage; if symptoms last less than 24 hours and there are no findings on brain imaging, it is considered a transient ischemic attack [1]. Stroke is a major cause of death worldwide with around 6.2 million deaths in 2015, and is one of the most common disabling diseases in the United States [1]. It estimated that a patient in the United States can end up paying around 4,800 dollars per month for poststroke care depending on the severity of the stroke [2].

Strokes can be further classified into ischemic or hemorrhagic. In a focal ischemic stroke, an embolus or thrombus can occlude a specific vessel within the brain causing symptoms depending on the location and the area of the brain that is supplied by that vessel. One major vessel of the brain is the posterior cerebral artery (PCA), which along with the vertebral arteries and basilar artery help supply the posterior circulation of the brain [1]. The major structures that are supplied by these vessels are the cerebellum, midbrain, pons, medulla, and, ultimately, the occipital lobe [1].

Posterior circulation strokes involve infarction occurring in the vertebrobasilar arterial system, which in contrast to anterior circulation strokes, can be challenging to diagnose and manage due to variable anatomy and nonspecific presenting symptoms [3]. This is especially true for strokes of the PCA, which despite being responsible for between 5% and 10% of acute ischemic strokes have not been studied as extensively as those of other vascular territories [3,4]. The most common symptoms are nonspecific and include headache and visual impairment [3]. The specific visual defect or dysfunction depends on the size and location of the infarct [5]. Here, we report a unique case of a left PCA stroke following diesel vapor and exhaust exposure, resulting in headache and contralateral monocular hemianopia.

## Case presentation

A 69-year-old male with a past medical history of tobacco use disorder presented to the emergency department with a one-week history of worsening headache and right temporal vision loss. The patient worked as a boat mechanic, and stated that one week ago he was working on a boat engine that was leaking diesel fuel. He worked on the engine for several hours in an enclosed room with it running intermittently. Shortly after leaving work, he noticed a throbbing, dull headache located across his forehead and temporal region, as well as loss of peripheral vision in his right eye. At presentation, the patient described that the headache and vision changes had worsened, but denied any focal neurological deficits. He stated that he first went to an urgent care facility where he was diagnosed with sinusitis and prescribed amoxicillin, which did not relieve any of his symptoms.

In the emergency room, the patient was initially hypertensive with a blood pressure of 181/92 mmHg, but it improved to 141/80 mmHg without medical intervention. Neurologic examination revealed a right monocular temporal hemianopia upon visual field confrontation, resulting in an NIH stroke score of one. The patient was admitted for further evaluation of his headache and peripheral vision loss of the right eye.

**Figure 1:**
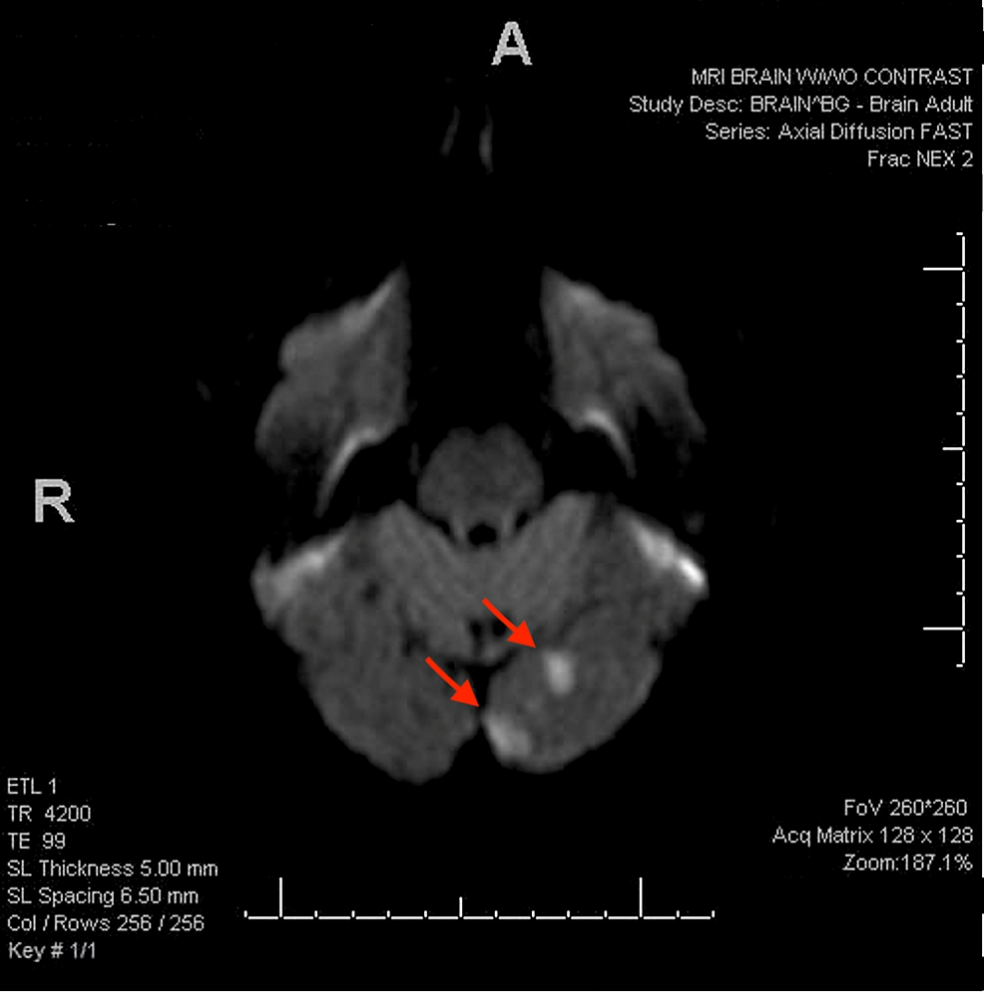
MRI of the brain demonstrating multifocal acute infarcts in the left occipital lobe. MRI: magnetic resonance imaging

**Figure 2:**
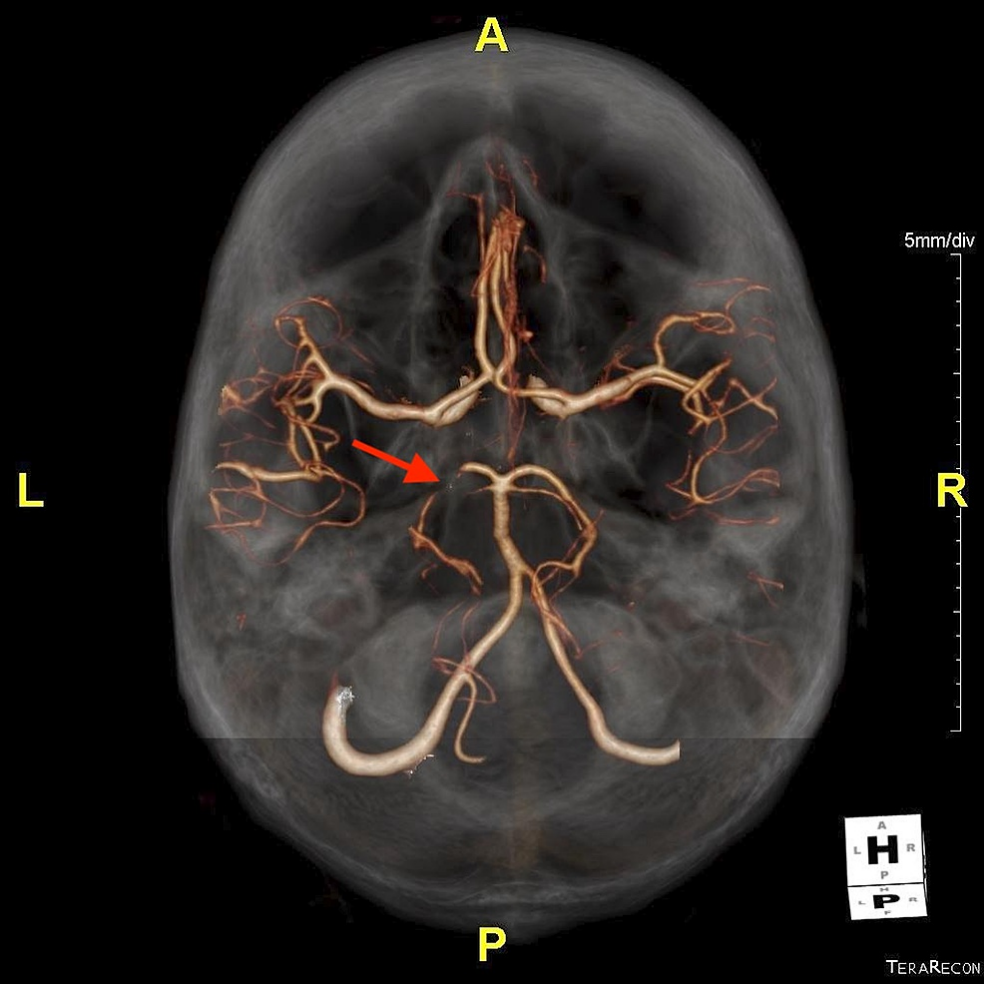
CT angiogram of the head demonstrating severe stenosis of the proximal left PCA. CT: computed tomography; PCA: posterior cerebral artery

On admission, magnetic resonance imaging (MRI) of the brain showed multifocal acute infarcts in the left occipital lobe, indicating left PCA disease and no hemorrhagic transformation (Figure 1). Given the signal on diffusion and enhancement, it was estimated the infarct was between three and seven days old. Computed tomography (CT) angiogram of the head pre-contrast demonstrated a small area of infarct in the left occipital lobe, corresponding with the MRI findings (Figure 2). After contrast administration, the left PCA demonstrated severe stenosis in the P1 segment. The distal branches of the PCA were still opacified and the posterior communicating arteries were faintly seen. This demonstrated severe stenosis of the PCA.

 

There was no indication for angiography or intervention due to the time that had passed since the estimated occurrence of the PCA infarct. A cardiac workup including electrocardiogram and echocardiogram with bubble study were performed to monitor for causes of cardioembolic stroke, and these studies returned with no abnormalities. The patient was advised to obtain a loop recorder to monitor the presence of atrial fibrillation but the patient denied. Medical management for the infarction was recommended. Modifiable risk factors such as blood pressure, glucose, and cholesterol were addressed with the addition of clopidogrel, atorvastatin, and lisinopril.

## Discussion

PCA infarcts are particularly difficult to diagnose due to the nonspecific and often inconsistent symptoms among patients [6]. The most common symptoms associated with PCA infarcts include headache and homonymous hemianopia with macular sparing [6]. These symptoms are nonspecific and overlap with numerous often less emergent diagnoses such as migraine headaches, acute sinusitis, and multiple sclerosis. Patients presenting with these nonspecific symptoms and numerous risk factors for ischemic stroke such as occupational exposures, male gender, advanced age, and tobacco use disorder warrant emergent additional imaging to rule out a potential ischemic stroke. This patient presented to an urgent care facility due to the onset of headache and unilateral vision loss where he was incorrectly diagnosed with sinusitis and prescribed amoxicillin. In this case, the delay in proper imaging resulted in inadequate care and resultant multiple infarctions to the occipital lobe.

The visual defect seen in this patient’s infarction is inconsistent with the typical presentation of an occipital lobe infarction. According to the CT angiogram, the P1 segment was severely stenosed leading one to think that he would be more likely to develop symptoms of P1 syndrome, such as ataxia, hemiplegia, or hemisensory loss depending on the area of the brain that was affected [1]. Usually, it is occlusion of the P2 segment that causes infarction to the occipital lobe leading to visual deficits [1]. Furthermore, evidence from the MRI showed multiple areas of infarct in the occipital lobe which is suggestive of cardioembolic disease, with the most common cause of embolus formation in the heart being atrial fibrillation [7]. It is possible that an embolus propagated through the PCA and fragmented into the various deeper segments causing the patient’s visual deficit.

The phenomenon of monocular temporal hemianopia in a retrochiasmal lesion such as this occipital lobe infarction is extremely uncommon, only having been reported once before [8]. Typically, the visual defect associated with a retrochiasmal lesion is homonymous hemianopia, while the monocular findings such as those seen in this patient are more often associated with defects anterior to the optic chiasm, such as optic nerve damage, or optic chiasm compression [9]. This atypical finding could be due to patient error, or due to the insensitivity of visual field confrontation testing [10]. This unique presentation adds further evidence to support the possible clinical presentation of monocular hemianopia due to a retrochiasmal infarction. This suggests that PCA stroke should be added to the differential for patients presenting with this unique visual field defect.

It is interesting to note that the onset of the patient’s symptoms associated with his PCA infarct occurred on the same day as an exposure to a high amount of diesel exhaust fumes. It is well known that there is a strong association between air pollution and cardiovascular disease [11]. Diesel fuel is one of the most commonly used sources of fuel worldwide, yet the health impacts of acute and prolonged exposure to its exhaust are not completely understood [12-14]. It remains unclear whether cardiovascular events related to diesel exposures are caused by an acute effect on physiologic vascular mechanisms or by persisting effects due to repetitive exposure [13,14]. It has been shown that diesel exhaust fume affects specific steps in the cascade of atherosclerosis and exerts a systemic impact by impairing most of the known pathophysiologic backgrounds of atherosclerosis. This includes inflammation, thrombosis, fibrinolysis, heart rate variability, increased thrombogenicity, platelet activation, endothelial cell functionality, and arterial stiffness, which ultimately culminate in cardiovascular system dysfunction [12-15]. Exposure to diesel exhaust particles has also been shown to significantly increase levels of certain inflammatory markers such as C-reactive protein and fibrinogen, while activities of anticoagulant proteins such as protein C and protein S decrease [12]. These factors further increase for thrombus formation, embolization, and possible ischemic cerebral infarcts. This patient’s occupation and the incident of acute exposure to diesel exhaust in an enclosed area may have contributed to accelerated arteriosclerosis in the PCA or possibly an undetected acute thrombosis, which led to the ischemic stroke. The stenotic vessel and possible decreased fibrinolytic activity from his acute diesel exposure may have exacerbated the patient’s decreased blood flow through the PCA, which culminated in the PCA infarct and resultant right-sided monocular temporal hemianopia.

## Conclusions

Obtaining a thorough patient history as well as gathering accurate and detailed information from a bedside physical examination is essential to differentiate a cerebrovascular accident from other etiologies such as migraine headache and sinusitis. PCA infarctions are particularly difficult to diagnose due to the nonspecific and unusual symptoms patients may present with such as monocular hemianopia. In the future, patients presenting with monocular hemianopia should raise suspicion for a PCA infarct and this should warrant further neurological imaging. Additionally, further investigation into the deleterious effects of short- and long-term diesel exposure on the cardiovascular system is necessary to elucidate its true role as a risk factor for cardiovascular and neurological events. With further research, better diagnostic approaches can be implemented to aid in the early diagnosis of PCA infarcts, and improved protocols can be put in place to care for patients in the emergent setting with a history of diesel exposure. This may aid in quicker and accurate diagnosis of PCA infarcts and improved long-term outcomes for patients.
